# Bridging Cancer Biology with the Clinic: Relative Expression of a *GRHL2*-Mediated Gene-Set Pair Predicts Breast Cancer Metastasis

**DOI:** 10.1371/journal.pone.0056195

**Published:** 2013-02-18

**Authors:** Xinan Yang, Prabhakaran Vasudevan, Vishwas Parekh, Aleks Penev, John M. Cunningham

**Affiliations:** Section of Hematology/Oncology, Department of Pediatrics, Comer Children’s Hospital, The University of Chicago, Chicago, Illinois, United States of America; University of Alabama at Birmingham, United States of America

## Abstract

Identification and characterization of crucial gene target(s) that will allow focused therapeutics development remains a challenge. We have interrogated the putative therapeutic targets associated with the transcription factor Grainy head-like 2 (*GRHL2*), a critical epithelial regulatory factor. We demonstrate the possibility to define the molecular functions of critical genes in terms of their personalized expression profiles, allowing appropriate functional conclusions to be derived. A novel methodology, relative expression analysis with gene-set pairs (RXA-GSP), is designed to explore the potential clinical utility of cancer-biology discovery. Observing that *Grhl2*-overexpression leads to increased metastatic potential *in vitro*, we established a model assuming *Grhl2*-induced or -inhibited genes confer poor or favorable prognosis respectively for cancer metastasis. Training on public gene expression profiles of 995 breast cancer patients, this method prioritized one gene-set pair (*GRHL2, CDH2, FN1, CITED2, MKI67 versus CTNNB1* and *CTNNA3*) from all 2717 possible gene-set pairs (GSPs). The identified GSP significantly dichotomized 295 independent patients for metastasis-free survival (log-rank tested p = 0.002; severe empirical p = 0.035). It also showed evidence of clinical prognostication in another independent 388 patients collected from three studies (log-rank tested p = 3.3e–6). This GSP is independent of most traditional prognostic indicators, and is only significantly associated with the histological grade of breast cancer (p = 0.0017), a *GRHL2*-associated clinical character (p = 6.8e–6, Spearman correlation), suggesting that this GSP is reflective of *GRHL2*-mediated events. Furthermore, a literature review indicates the therapeutic potential of the identified genes. This research demonstrates a novel strategy to integrate both biological experiments and clinical gene expression profiles for extracting and elucidating the genomic impact of a novel factor, *GRHL2*, and its associated gene-sets on the breast cancer prognosis. Importantly, the RXA-GSP method helps to individualize breast cancer treatment. It also has the potential to contribute considerably to basic biological investigation, clinical tools, and potential therapeutic targets.

## Introduction

One in eight women develops breast cancer during their lifetimes, the most common cause of malignancy in females. Although the majority of women now survive for many years after initial diagnosis and therapy, there is a need for individualized therapeutic decisions, as a significant subpopulation are at risk from metastatic breast cancer, and have a median survival time of 18–30 months [1]. Approaches to identify such individuals include the use of histologic grade, estrogen receptor status, and more recently tumor gene expression profiling. In addition, numerous genomic defects have been identified including gene deletions, translocations and locus amplification [2], [3]. Of these, a chromosomal region at 8q22.3 is of particular interest, with amplification and enhanced expression of several genes in this locus being implicated in poor treatment outcomes including chemoresistance, metastasis, and recurrence [4], [5].

We have become interested in exploring the role of the Grainy head-like 2 (***GRHL2***) transcription factor in mediating the poor outcomes observed in individuals with breast cancer who have amplification of the 8q22.3 locus. Preliminary data from our laboratory and others, suggests that it may have a role not only in normal epithelial ontogeny, but also in tumor progression and metastasis [4]–[6]. We thus theorize that, in the context of the molecular function of the gene(s) critical for tumor progression, such as *GRHL2*, creating an individualized profile of a patient is the key for translating cancer biology and informatics observations into clinical utility.

Genome-wide high-throughput technologies have enhanced our understanding of breast and other cancers. However, identified gene-sets have shown minimal overlap between various signatures, and lack the convenience and simplicity necessary for clinical application [7], [8]. In the case of transcriptomic microarray data, numerous prognostic gene signatures have been identified for breast cancer (reviewed by Joan Massagué [9]). Despite their predictive significance, the complexity and discrepancy of the signatures does not allow the easy extraction of biologically and therapeutically relevant and mechanistically-driven information.

Relative Expression Analysis (**RXA**) of gene pair(s) has been proposed to address the above deficiency (reviewed in [10]). RXA has a simplicity of practice and reproduction and is invariant to data normalization and parameter-fitting. The selected pairs of genes may not necessarily be ‘significant’, in terms of an arbitrary parameter in the analysis of primary human biological samples that is cohort- and threshold-dependent. The rationale for feature selection in this method is to use one gene as a “pivot” for another gene [10]. Initial studies using RXA have confirmed its ability to reveal novel gene pairs for cancer sample classification [11] and clinical diagnosis [12]. However, pure RXA is computationally intensive thus is limited to the study of biological mechanisms involving only two or three genes [13]. Most biological or clinical phenotypes, as results of multiple genes’ feed-forward and feed-back regulations, thus cannot be accurately elucidated by a RXA model.

We hypothesize that extending the RXA strategy from gene pair(s) to biological gene-set pair(s), Relative Expression Analysis with Gene-Set Pair (**RXA-GSP**), will not only allow novel insights into mechanism-dependent disease-associated molecular functions, but enhance the robust nature of the identified classifiers of disease prognosis. By feature selection among each biologically relevant gene-set, the RXA-GSP strategy is superior to previous RXA methods because it can interrogate unlimited number of genes as a gene-set pair. More importantly, RXA-GSP strategy derives individualized indexes based on relative expressions of selected gene-set pair for an unbiased evaluation, as it allows us to merge samples from multiple laboratories using different platforms without additional data preprocesses. Even more, we expect that RXA-GSP will facilitate the use of cancer biology discovery to make individualized prognostic and therapeutic decisions, as an experimental pre-definition of two gene-sets allows us to focus on biologically and therapeutically relevant and/or mechanistically driven information. In this manuscript, we demonstrate the identification of a new biomarker, *GRHL2*, together with other six genes to play a breast cancer prognostic role, using RXA-GSP.

## Results

### High Expression of *GRHL2* Contributes to Increased Metastatic Potential (Fig. 1, Table S1)

**Figure 1:**
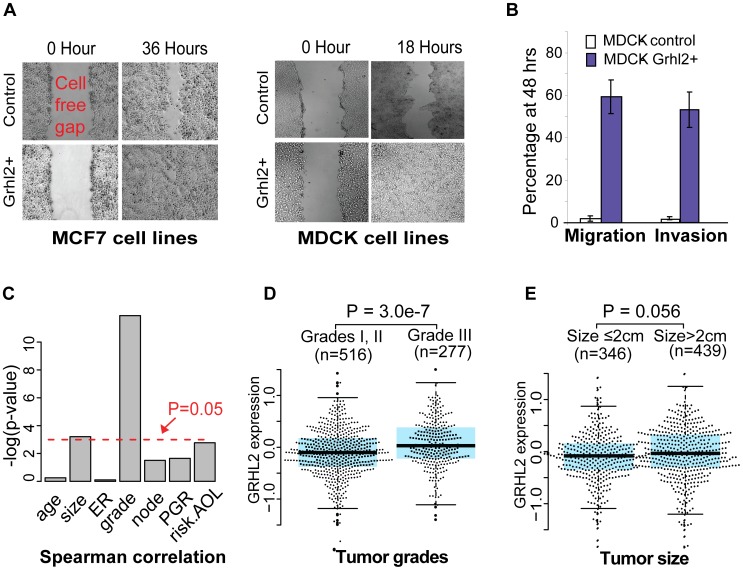
High level expression of *GRHL2* contributes to increased metastatic potential. **A**) Temporal comparison of wound repair in MCF7 and MDCK cells in which *Grhl2* expression is baseline (Control) or enforced (*Grhl2*+). **B**) Boyden chamber migration and invasion assays on control and *Grhl2* overexpressed (*Grhl2*+) stable clones for MDCK cells. Fraction of seeded cells that migrated to the bottom chamber at 48 hours is dramatically higher in *Grhl2*+ clones than in the controls. Data represent mean±SD from triplicates. **C**) Continuous expression measurement of *GRHL2* is significantly correlated with the histological tumor grade and the measurement of tumor size in mm (p = 6.8e–6 and 0.041, respectively, Spearman measurement). **D**) *GRHL2* expression is significantly higher in patients with grade III breast tumors when compared with grade I or II tumors (two-sided t-test with unequal-variance). **E**) *GRHL2* expression reaches significance in patients with large tumors at diagnosis (>2 cm) when compared to smaller tumors (two-sided t-test with unequal-variance).

The human *GRHL2* gene is localized on chromosome 8q22.3. It is amplified and overexpressed in breast cancers [5] and is associated with a poor prognosis [4]–[6]. The human *GRHL2* gene is localized on chromosome 8q22.3. It is amplified and overexpressed in breast cancers [5] and is associated with a poor prognosis [4]–[6]. Recently, Leth-Larsen et al. have found that high level expression of *Grhl2* is associated with an increase of tumor invasion in a mouse model *in vivo* [6]. Cieply *et. al.*, however, reported *Grhl2* downregulated specifically in the tumor initiating cells compared with other tumors [14]. To confirm that *Grhl2* contributes to increased metastatic potential in epithelial cells, we generated several stable *GRHL2* overexpression (**G+**) clones for MCF7, a human breast adenocarcinoma cell line, and MDCK, a canine kidney cell line. The latter is a cellular model widely used to study cell polarity, intercellular adhesion and cellular migration. In both models, wound repair, an event known to correlate with enhanced tumorigenicity was more effective in G+ cells when compared to control cells (**Fig. 1A**). In addition, we observed a dramatic increase in migration and invasion potential in G+ cells (**Fig. 1B**).

To confirm that higher *GRHL2* expression is an unfavorable metastatic factor related to histological grade of the breast tumor, we evaluated a collection of six independent datasets pertaining to primary human breast cancer [15], containing 947 independent primary breast tumor samples [16]–[21] (**Fig. 1C–1E**). We first checked the prognosis of *GRHL2* expression using 509 samples of untreated patients. As listed in **Table S1,** the single variable analyses of distant metastasis-free survival (**DMFS**) for *GRHL2* and four clinical prognostic factors (ER status, lymph node status, tumor size and tumor grade) are significant (log-rank tested p<0.05). Interestingly, higher *GRHL2* expression is significantly correlated with two unfavorable prognostic characters - progressive tumor grade III and large tumor size (>2 cm) at the time of diagnosis (**Fig. 1C–1E**). Multivariate Cox proportional hazards analysis of these five significant DMFS factors indicated independence between *GRHL2* expression and the other three clinical characters but not the histological grade of the tumor. In addition, the continuous expression levels of *GRHL2* itself demonstrated significant response in DMFS (p = 0.041, **Table S1**). However, dichotomized *GRHL2* expression based on its median expression value could not predict patients’ outcome significantly (p = 0.5), restraining its personalized clinical application.

### Twelve *GRHL2*-mediated Prognostic Marker Candidates (Table S2)

To explore a reproducible prognostic model, we focused on *GRHL2* together with seven *GRHL2*-mediated “poor-prognosis” candidates including N-cadherin *(CDH2), ACTA2, FN1, TGFB3, CITED2, RHOA;* the proliferation marker *MKI67*; and four “good-prognosis” epithelial markers including E-cadherin (*CDH1),* α-catenin *(CTNNA1),* β-catenin *(CTNNB1)*, and *α*-T-catenin (*CTNNA3*).

These genes were selected through the evaluation of the literature for genes implicated in cancer metastatic prognosis, specifically the epithelial to mesenchymal transition or EMT phase, and experiments that explored potential *Grhl2*-associations in terms of both mRNA and protein expression (**Table S2**), using murine and human breast adenocarcinoma cell lines (4T1 and MCF-7), as well as, a human breast epithelial cell line (MCF10A). When comparing G+ cell with control cell lines in an *in vivo* mouse model, all poor-prognostic candidates had higher levels of expression while good-prognostic candidates had diminished expression using MCF10 cell lines. Three biological repeats were performed to confirm these observations. Using MCF7 and 4T1 cell lines, similar properties were observed for all genes except the *MKI67* gene (**Table S2**).

The 4T1 mammary carcinoma is a transplantable tumor cell line that is highly tumorigenic and invasive, and can spontaneously metastasize from the primary tumor in the mammary gland to multiple distant sites [22]. The low-tumourigenic breast cancer MCF-7 cell line is widely used as models for the study of cell polarity, intercellular adhesion and cellular migration. The breast epithelial MCF10A cell line is widely used for modeling induced proliferation and invasion in breast cancer cells [23], [24]. Using 4T1 cell line, the miR-155 promotes macroscopic tumor formation in the lung with a significantly upregulation of *Grhl2* [25]. Using a mouse model of 4T1 and MCF-7 cell lines, we have demonstrated EMT-mediated enhanced metastatic spread of *GRHL2*-overexpressing cells in a mouse model of breast cancer (data not shown). Specifically, we explored the switch in cadherin gene expression that accompanies EMT, from membrane E-cadherin, a widely acting suppressor of invasion and growth of epithelial cancers [26] to N-cadherin which promotes cellular invasion, with a consequent increase in metastatic potential [27]. Similarly, three catenin homologues are downregulated in invasive tumor cells compared with the normal controls [28], [29]. Indeed, previous studies of *Twist*, another transactivator regulating EMT, demonstrated that forced expression of *Twist* results in coordinated down-regulation of *CDH1* and *CTNNB1*, while *FN1*, *CDH2* and *MKI67* are up-regulated [30]. Similarly, we chose *RHOA* [31], *MKI67* [32], *CITED2* [33], *ACTA2* [34], *FN1* [35] and *TGFB3* [36], as all have been implicated in EMT and highly metastatic breast cancer. It is important to note that when comparing expression in G+ cell lines with controls, we grouped induced genes into the “poor” prognostic candidate gene-set and those genes with diminished expression into a “good” risk gene-set.

### Relative Expression Analysis with Gene-set Pairs (RXA-GSP) on Gene Expression of 995 Patients for Distant Metastasis-free Survival (DMFS) (Fig. 2A)

**Figure 2:**
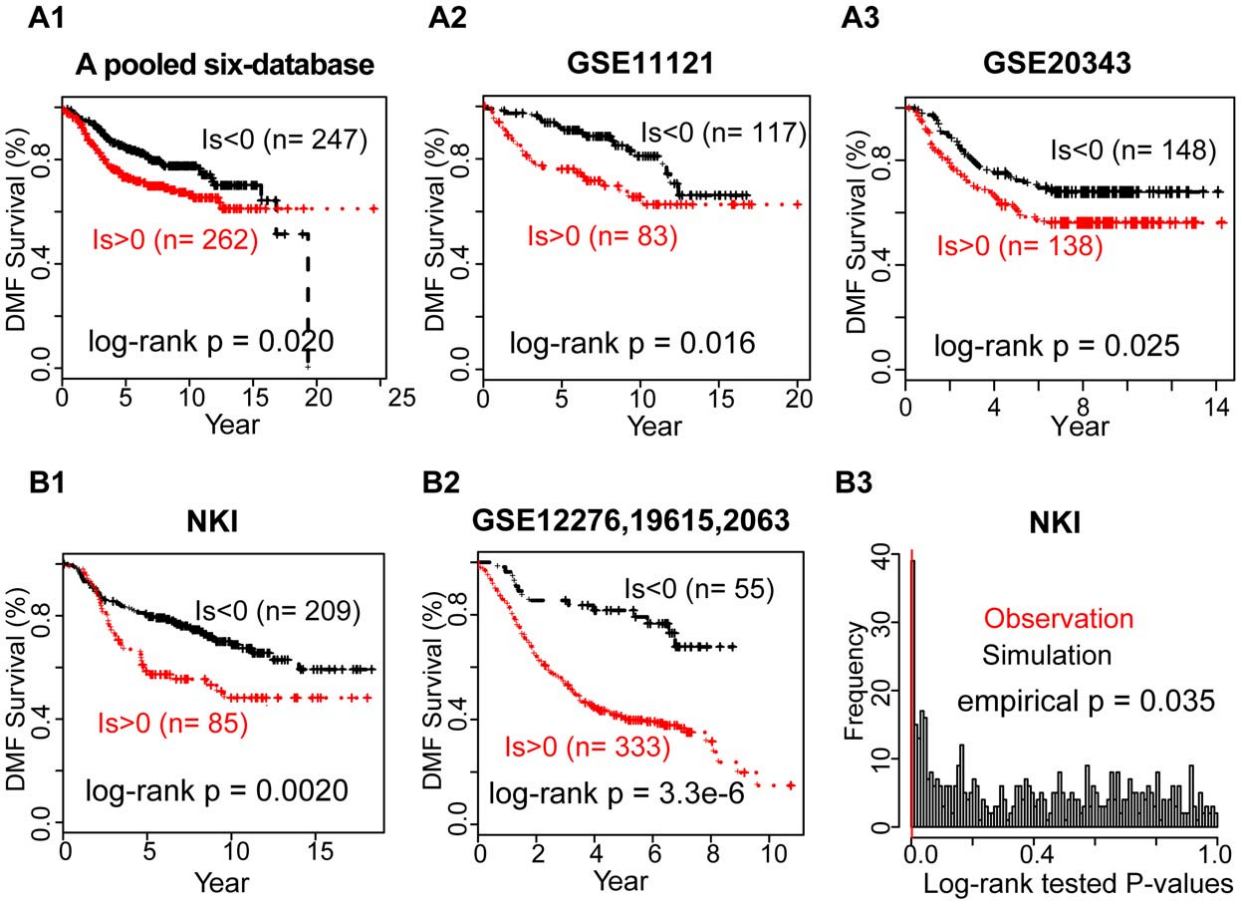
A gene-set pair shows significant prognosis for breast cancer distant metastasis-free survival (DMFS). The positive individualized prognostic index, i.e. relatively higher expression of the poor-prognosis gene-set (*GRHL2, CDH2, FN1, CITED2, MKI67*) compared to the good-prognosis gene-set (*CTNNB1, CTNNA3*), significantly predicts unfavorable DMFS in the training datasets (**Panels A** 1–3). Importantly, the identified GSP shows significant prognosis in two independent validation datasets (**Panel B**), theoretically (**Panels B** 1–2) and empirically (**Panels B** 3).

Based on the selected and evaluated marker candidates, eight poor-prognostic and four good-prognostic, we evaluated the DMFS for all 2717 possible GSPs (**Eq. 1).** Each GSP comprises two or more poor-prognostic and two or more good-prognostic candidates. Examining all combinations of the biologically-driven GSPs on gene expression profiles of human samples allows us to select the most clinically relevant GSP. We identified significantly prognostic GSP(s) from a collection of 995 primary breast cancer patients (three computational cohorts from eight independent datasets [16]–[21], [37], [38], Affymetrix platform, **Fig. 2A** **,** **Table 1**). The voting of *GRHL2* in a selected GSP illustrates its clinical impact, as these 2717 GSPs include 1397 GSPs with *GRHL2* and 1320 GSPs without *GRHL2*. The positive individualized prognostic index (**Methods, Eq. 2**) of one GSP, i.e. relatively higher expression of the poor-prognosis gene-set (*GRHL2, CDH2, FN1, CITED2, MKI67*) compared to the good-prognosis gene-set (*CTNNB1, CTNNA3*), was significantly associated with an unfavorable DMFS in all training datasets (Hazard ratio>1 and log-rank tested p<0.03 across three computational training cohorts, **Fig. 2A** **Panels 1–3**). Notably, this GSP was identified as the only single significant predictor among all 2717 tested GSPs using the Liptak-Stouffer method [39] (meta-analysis p = 0.0018, Bonferroni adjusted p = 0.005, **Eq. 3, Data S1**).

**Table 1:** Summary of microarray expression profiles and sample information.

	Training					Validation1	Validation2				
	Pooled six$		GSE11121		GSE2034	NKI	GSE12276	GSE19615		GSE2063	
Number of patients with outcome	509	200		286		295	192		115		81
Censored distant metastasis: 0/1	374/135	154/46		179/107		194/101	10/173		101/14		54/27
ER:−/+	154/350	70/155*		77/209		69/226	192/0		45/70		35/46
LN:−/+	353/151	200/0		286/0		151/144	192/0		62/53		0/81
DMFS year (mean ± sd)	6.9±4.6	7.8±4.2		6.5±3.5		7.3±4.1	2.2±1.9		4.9±2.1		5.4±2.4
Grade:1/2/3	62/171/158	29/136/35		7/42/148*		75/101/119	NA		23/28/64		NA
Age (years, median ± sd)	52±12#	60*		52±12*		44±5.5	50±13		53±12		54±13
Tumor size (mm, mean ±sd)	26.2±13#	21±10		NA		22.5±8.9	NA		14±1.2		37±18
Platform	Affymetrix Hgu133a					Agilent	Affymetrix Hgu133-plus2				Affymetrix Hgu133a
Reference	BMC Genomics2008 [15]	Cancer Res 2008 [37]		Lancet 2005 [38]		N Engl J Med 2002 [47]	Nature 2009; 459 (7249):1005–9 [40]		Nat Med 2010; 16(2):214–8 [5]		Nature 2005; 28;436 (7050):518–24 [41]

$ :Van Vliet *et. al*. have described a pooled six-dataset [15]. Five components were collected from the NCBI’s Gene Expression Omnibus (GEO, http://www.ncbi.nlm.nih.gov/geo/) with the following identifiers: GSE6532 (GSE2990), GSE3494, GSE1456, GSE7390 and GSE5327. An additional dataset was collected from ArrayExpress (http://www.ebi.ac.uk/, identifier E-TABM-158).

* :information derived from the manuscript but not publicly available.

# :Summary of subset of the 509 patients with available information [15].

### Evaluation the DMFS Prognostic Power of the Identified RXA-GSP Using 683 Independent Patients (Figs. 2B, 3)

More importantly, the identified GSP shows significant prognosis in the NKI validation dataset, theoretically (p = 0.002, log-rank test) and empirically (p = 0.035) (**Fig. 2B** **panels 1 & 3**). Using the profiles of another 388 independent patients collected from three studies [5], [40]–[42], the relative expression of this GSP dichotomized tumors into two prognostic groups for metastasis-free survival (log-rank tested p = 3.3e–6, **Fig. 2B** **panel 2**). Note that RXA-GSP strategy derives prognostic index based on individual profiles of selected gene-sets, allowing merging samples from multiple laboratories using different platforms without additional data preprocesses for an unbiased evaluation (**Data S2**). As the GSP was derived from a large cohort with an average survival time around 7 years, it failed to stratify patients in GSE12276 with a mean survival time around 2 years, predicting all cohort to be poor outcome (**Data S2**). Additionally, the majority of the patients in the pooled three-dataset were assigned to have poor outcome (Is>0), which coordinates with the fact that the overall survival time of patients in the pooled three-dataset is shorter than that in the training datasets (**Table 1**). Overall, these results support the robustness and the potential of individualization of prognosis using RXA-GSP strategy.

**Figure 3:**
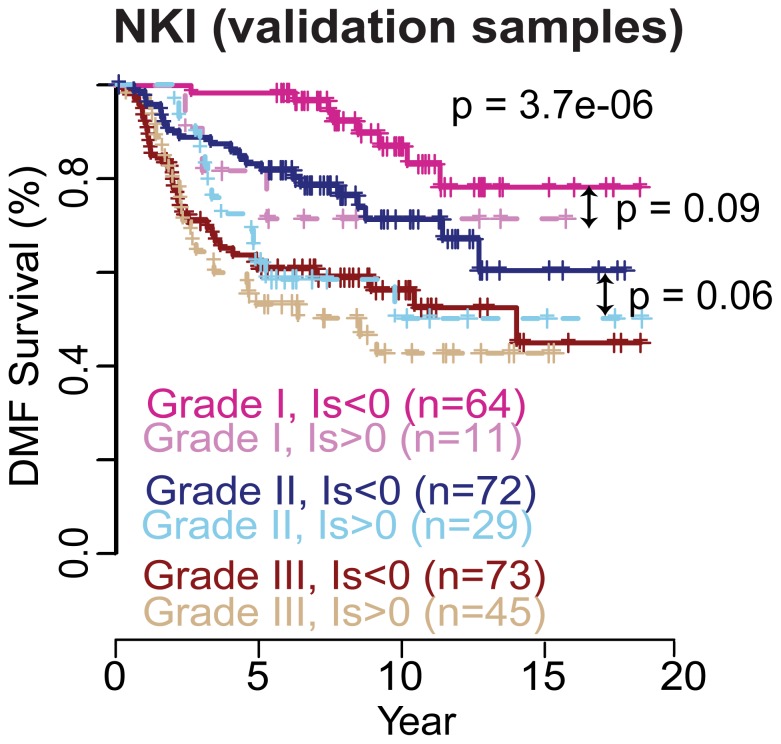
Multivariate analysis of the model combing GSP and tumor grade improves the prognosis of DMFS for both. The multivariate analysis combining the GSP and tumor grade shows that, in each of the three tumor grade sub-cohorts, the positive individualized prognostic index confers poor DMFS. The multivariate analysis p-values are 0.055, 0.024 and 0.018 and hazard ratios are 1.5, 1.6 and 0.4 for GSP, tumor grade III and tumor grade I, respectively; the p-value for the overall model is 2.7e–6.

To discover the clinical potential of the seven markers consisting of the identified GSP, we subsequently conducted functional analysis through the use of IPA (Ingenuity® Systems, www.ingenuity.com), and a literature review. Evidence supports crucial roles of these markers in breast cancer development, diagnosis (**Fig. S1, Table S2**), and targeted therapy (**Table S3**). Additionally, this identified GSP is independent of most traditional prognostic factors while significantly associated with the histological grade of the tumor (p = 0.0017), among the 295 NKI validation patients (**Table 2**). Multiple variable proportional-hazards analysis of DMFS showed an improvement of prognostication by combining GSP and tumor grades (log-rank p = 3.7e–6 for the multivariate model, **Fig. 3**; p = 0.002 for GSP only, **Fig. 2B** **panel 1**; and p = 4.9e–6 for tumor grade only, plot not shown). Additionally, the GSP could further stratify DMF survival for patients with low- or intermediate-grade tumors (log-rank p<0.1), by univariate analysis of the corresponding histological sub-cohorts.

**Table 2:** Association between clinical characteristics and the identified gene-set pair (GSP).

Characteristic	Good-prognosis (N = 209)	Poor-prognosis (N = 85)	P value (FET)	Characteristic	Good-prognosis (N = 209)	Poor-prognosis (N = 85)	P value (FET)
	No. of patients (%)				No. of patients (%)		
Age			0.11	St Gallen			0.14
<40 yr	38 (18)	24 (28)		0 (inter/high risk)	190 (91)	52 (96)	
40–44 yr	61 (29)	24 (28)		1 (low risk)	19 (9)	3 (4)	
45–49 yr	77 (37)	21 (25)		Estrogen-receptor status			0.23
>50 yr	33 (16)	16 (19)		Negative	45 (22)	24 (28)	
No. of positive nodes			0.42	Positive	164 (78)	61 (72)	
0 (none)	102 (49)	48 (56)		Mastectomy surgery			1
1–3	80 (38)	26 (31)		No	114 (55)	46 (54)	
>4	27 (13)	11 (13)		Yes	95 (45)	39 (46)	
Tumor diameter			0.37	Chemotherapy			0.51
<20 mm	113 (54)	41 (48)		No	128 (61)	56 (26)	
>20 mm	96 (46)	44 (52)		Yes	81 (39)	29 (14)	
Histologic grade			**0.0017**	Hormonal therapy			0.45
I (good)	64 (31)	11 (13)		No	178 (85)	76 (89)	
II (intermediate)	72 (34)	29 (34)		Yes	31 (15)	9 (11)	
III (poor)	73 (35)	45 (53)					

FET, the Fisher’s exact test.

Patients’ clinical information was downloaded from NKI (http://bioinformatics.nki.nl/data.php).

### Six Putative *GRHL2* Targets (Figs. 4, S2)

**Figure 4:**
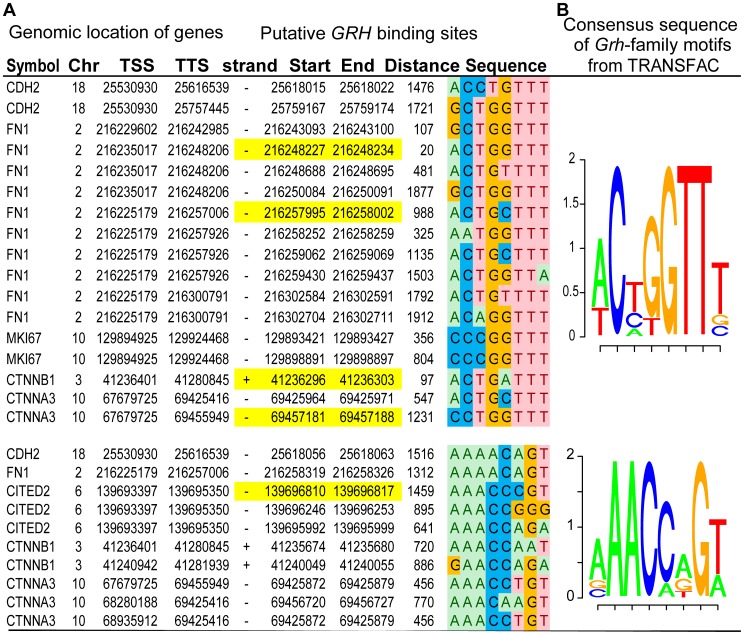
Putative biding sites at the promoter region of each of the six genes (*CDH2, FN1, CITED2, MKI67, CTNNB1* and *CTNNA3*) and the known consensus *Grh* binding motifs. A) Genomic regions identified to be putative binding sites of *GRHL2*, using the hg19 build 37 of the human genome. Cross-species conserved regions are highlighted in yellow and are demonstrated in **Fig. S2**. **B)** Visualization of the position weight matrices of the reverse-complementary DNA sequence motifs of Grainyhead (*Grh*) factors (TRANSFAC database [43]).

Our results have shown the expressional association and clinical relevance of six genes (*CDH2, FN1, CITED2, CTNNB1,* and *CTNNA3*) together with *GRHL2* for breast cancer metastases (**Table S3**). There is no published genome-wide experiments pertaining to *GRHL2* binding. To address the question whether these six genes are potential targets of *GRHL2*, we searched for a consensus DNA target sequence of Grainyhead (*Grh*)-family factors described in TRANSFAC database [43] (**Fig. 4B**), over the 2 kbp 5′ untranslated region (5′UTR) of these six genes. Analysis of the regulatory regions of the these six genes revealed at least one highly matched *Grh*-binding site for each (**Fig. 4A**), suggesting these six genes as putative targets of *GRHL2*. Because functionally important sequences are frequently conserved between evolutionarily distant species, we did further genomic comparison for the identified sequences [44]. Among these putative binding sites on human genome, all except *MKI67* are cross-species conserved (**Fig. S2**), suggesting a potential evolutionarily constrained role. Further biological validation, e.g. using ChIP-qPCR or a related technique, could determine whether these genes are indeed *GRHL2* direct targets.

## Discussion

This study demonstrates a novel strategy to use transcriptomic data for elucidating the genomic impact of a new prognostic transcription factor, *GRHL2*, on breast cancer metastasis-free survival. We suggest that our new method, relative expression analysis with gene-set pairs (RXA-GSP), shows promise for individualized prognosis as it is driven by biomolecular hypotheses, is anchored in biological experiments, and is validated using clinical data. The merits of RXA-GSP lie in: i) its simplicity for physicians to use expression measurements of limited number of genes to generate clinical prediction that may guide decisions; ii) its avoidance of arbitrary thresholds for the continuous expression variables; iii) the model’s structure that explains the prognostic effects of a critical gene-set or pathway. More experimental evidence using more ‘anchor’ genes will allow us to improve its reliability.

In this study, the controversial expression of *Mki67* between *Grhl2* overexpressed cancer cell lines and *Grhl2* overexpressed breast epithelia cell line might be due to the difference in cell cultures or cell context-specific effects. The lack of cross-species conservation for the putative *Grhl2* binding regions further indicates that *MKI67* might be an indirect *GRHL2* target. By alternatively assigning *MKI67* to the “good-prognosis” candidates, however, we could not identify any GSP to meet the three significance criteria applied. These results suggest that an adverse prognostic effect of *MKI67* in breast cancer metastasis is co-regulated by other complex mechanisms besides *Grhl2*-mediation, or is triggered in different stages between tumorigenesis and metastasis.

In the identified GSP, all six genes other than *GRHL2* have at least one promoter region matching to the *Grh*-binding consensus motif. This result combined with the *GRHL2*-dependent expression and clinical co-function indicates that the six genes are putative targets of *GRHL2*. However, more biological experiments are needed to validate an extra assumption that *GRHL2* plays a role as a tumor metastatic driver in breast cancer. To further evaluate the clinical impact of these seven identified genes as a gene-set pair, more cohorts could be tested on mRNA and protein levels.

Taken together, the tumor proliferation marker *MKI67* and a new prognosis marker *GRHL2* collaborate with several mesenchymal markers as a “poor-prognosis” gene-set, while two epithelial markers perform as a “good-prognosis” gene-set. No single gene significantly confers prognosis across all studied cohorts. However, the relative expression of the identified gene-set pair significantly associates with the histological grade of the breast tumor and generates significantly prognostic stratifications for distant metastasis-free survival. In summary, this study demonstrates a novel strategy of measuring gene expression that is helpful to derive prognosis and is founded in biological significance. We expect that this method will have application in many datasets, such as protein expression, gene copy number, or metabolomics.

### Conclusion

Gene signatures derived from microarrays have significant prognostic power. However, identification and characterization of crucial gene targets that will allow focused therapeutics development remains a challenge. Transcription factors represent attractive targets to alter cancer development/progression. Therefore, we propose a novel methodology which is designed for bridging cancer biology of attractive gene(s) with the clinic. This novel feature selection method makes two specific contributions to translational cancer research. First, there is a significant need for valid prediction models that are based on underlying biological mechanisms. As an *in vivo* experiment-anchored mechanism, all gene markers were associated with *Grhl2* expression in mouse models of murine and human breast cancer/epithelia cell lines. Second, this method is a crucial step toward clinical utility. It summarizes the individualized relative expression between gene-set pairs, thus tolerating the diverse noise and differences observed from multiple technologies and laboratories. Using a collection of 1678 independent human breast cancer samples, we identified and validated a new *GRHL2*-mediated gene-set pair that could effectively stratify patients showing significant differences in metastasis free survival. Thus, this research has the potential to contribute considerably, not only to basic biological investigation but also to its translation into clinical diagnostic and prognostic tools.

## Materials and Methods

### Biological Experiments and Hypothesis Generation

To confirm that *Grhl2* overexpression (G+) increased migration and invasion potential, the scratch wound healing assays and Boyden chamber migration and invasion assays were used for MCF7 cells and MDCK cells. When comparing both murine and human breast cancer G+ cell lines with control cells, the “poor-prognosis” marker candidates that had higher expression in G+ cell lines were grouped together while the “good-prognosis” candidates that had lower expression in G+ cells were grouped together. The study was approved by the Institutional Animal Care and Use Committee at the University of Chicago (ACUP number: 71819).

#### Cell cultures

4T1 mouse cell line [45] was kindly provided by Dr. Fred R. Miller at the Wayne State University. As described in the original publication [45], these 4T1 cells were cultured in Dulbecco’s Modified Eagle Medium supplemented with 10% Fetal Bovine Serum and antibiotics (100 units/ml penicillin and 100 µg/ml streptomycin) at 37°C in a humidified atmosphere containing 5% CO_2_. MCF7 (American Type Culture Collection - ATCC, Catalog No. HTB-22), MDCK (ATCC, Catalog No. CRL-2936) and MCF10A (ATCC, Catalog No. CRL-10317) cell lines were cultured as suggested by American Type Culture Collection (ATCC).

#### *Grhl2*-overexpressing (G+) experiments

Using hemagglutinin (HA)-tagged murine full-length *Grhl2* cDNA, we generated three stable overexpression clones for *Grhl2* in the murine and human breast cancer cell lines (4T1 and MCF7, normal two-dimensional cell culture, respectively), as well as the non-tumorigenic epithelial cell line MCF10A in three-dimensional cell culture (cultured in Matrigel). MCF10A cell line was used as we and others have reported that MCF10A G+ promotes adverse prognostic tumor features including a mesenchymal phenotype [46], increased cell motility and invasiveness [23]. The full-length mouse *Grhl2* cDNA clone was purchased (Open Biosystems, Catalog No. MMM1013-9201448), linked in frame with an HA-tag sequence at the 5′ UTR end, and subcloned into pcDNA3.1 plasmid (Invitrogen, Catalog No. V790-20).

### Microarray Profile Collection and Analysis

Twelve published, author normalized, independent microarray datasets pertaining to human primary breast cancer were investigated [5], [16]–[19], [21], [37], [38], [40]–[42], [47] (**Table 1**), since we assumed that the relative expression analysis tolerates the variance causing by multiple laboratories and platforms. Among eight training sets, six breast cancer datasets (totaling 947 samples) were collected and processed by NKI (http://bioinformatics.nki.nl/data.php) [15] (hereafter referred to as “pooled six-dataset” for simplification). In addition, two large datasets of untreated primary breast cancer patients were downloaded from GEO by the access numbers GSE11121 [37] and GSE2034 [38]. Validation was based on the well-assessed, whole-genome gene expression of 295 primary breast carcinomas [47] that was downloaded from NKI (hereafter referred to as “NKI dataset”). Additional expression profiles of another 388 patients were collected from three recent studies of breast cancer [5], [40], [41] (referred to as “pooled three-dataset”). Their metastatic follow-up information was collected from GEO or from a recent meta-analysis [42].

Gene expression data analysis was performed by Bioconductor (http://www.bioconductor.org) in R (version 2.14.0). For genes with multiple designed probe(set)s, the one with the highest across-sample variance was selected to represent its targeting gene. Spearman nonparametric test was carried to evaluate the correlation between *GRHL2* expression and seven known prognostic factors. The R package *Survival* was used to produce Kaplan-Meier plots and to carry out log-rank tests.

### Relative Expression Analysis (RXA) with Gene-set Pair (GSP)

To build a robust prognostic predictor while taking the knowledge of tumor biology, we proposed a novel relative expression method (RXA-GSP) based on the biological hypothesis and experiments. The proposed individualized index (**Eq. 2**) dichotomized patients into two prognostic groups. A positive index indicated poor prognosis and a negative value indicated good prognosis.

#### GSP

There are two predicted prognostic gene-sets, the “poor prognostic set’ (*Sp*) consisted of markers (*M+*) that had higher expression in G+ than in control cells; while the “good prognostic set” (*Sg*) contained the markers (*M–*) that had lower expression in G+ than in the control cells. Let any one *Sp* together with one *Sg* comprise a GSP, and each gene-set comprising of at least two markers, we built the gene-set pairs. Given the cardinality (count of genes) of each gene-set *S* to be |*S*|, the number of overall GSP combinations *N* was calculated as:
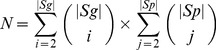
(1)


#### RXA-GSP

We then built an individualized prognostic index for each sample *s*. This index was defined as the median expression of the *M+* markers subtracted by the median expression of the *M–* markers. Thus, a positive index indicated poor prognosis and a negative value for good prognosis.
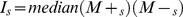
(2)


### Identification and Validation Prognostic Gene-set Pair

In each computational training cohort (pooled six-dataset, GSE11121, and GSE2034 in **Table 1**), Cox regression was used to estimate the prognostication of DMFS for every GSP. Then a meta-analysis was conducted on the cohort-dependent P-values using the Liptak-Stouffer method [39], a weighted sum of the inverse normal transformation of the P-values with weights determined by sample sizes. 
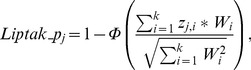
(3)


where the weighting factor *W* corresponds to the sample size in an individual study, *k* corresponds to the number of training cohorts; *_Zi_ = Φ^−1^(1 − p_i_)*, *p_i_* is a P-value for the *ith* cohort with *W_i_* patients, and *Φ* and *Φ^−1^* denote the standard normal cumulative distribution function and its inverse. To prioritize prognostic GSPs, we assessed the combinations of all possible GSPs and selected the features using the following criteria.

1. The GSP was significant in meta-analysis (false discovery rate (FDR)<5%, Bonferroni adjustment for multiple comparisons), ensuring the statistical significance across cohorts.
2. In each cohort, the individualized index (**Eq. 2**) of the GSP stratified samples into two balanced groups (each group had at least 20% of the cohort size), ensuring the clinical feasibility.
3. Given the individualized index defined in **Eq. 2**, the GSP predicted a unfavorable DMFS in each cohort (with a hazards ratio larger than 1 and its unadjusted, cohort-dependent, log-rank tested p<0.05) to satisfy the biological assumption about “poor”- and “good”-prognosis.

Finally, the selected models were validated in the patient- and technology-independent NKI dataset [47] after calculating the corresponding individualized prognostic index of the identified GSP. Furthermore, the prognostic significance of this GSP was further evaluated by permutation resampling the same size of genes (5 in one set and 2 in another set) to calculate an empirical p-value. This empirical p-value is more severe than log-rank tested p-values, as random signatures might also predict breast cancer outcome [7]. Similarly, the selected model was further validated in the a pool of another three independent datasets [5], [40], [41].

### Searching for Consensus DNA Binding Sequence of *Grh* Factors for Six Genes in the Identified GSP Besides *GRHL2*

The *Grh* factors binding motif (ACYGGTTT), its reverse-complement (AAACCRGT), and their position weight matrices (PWMs) are described in TRANSFAC database [43]. We used Bioconductor packages *TxDb.Hsapiens.UCSC.hg19.knownGene* (v.2.8.0) and *BSgenome.Hsapiens.UCSC.hg19* (v.1.3.19) to extract the 2000 bp upstream sequence for each of the six genes. We matched these two reversely matched PWMs to each of the promoter sequence, using Bioconductor package *Biostring* (v.2.26.2). The predictive binding sites were the regions having a minimum score for counting a match to be 85% of the highest possible score [48]. We then removed the repeated hits caused by transcript overlap. The cross-species conservation of identified binding regions was assessed using UCSC genome browser (http://genome.ucsc.edu).

## Supporting Information

Figure S1 **Clinical impacts of the seven genes in the identified GSP.** The solid lines link genes with their clinical roles and biological functions, using the Ingenuity® IPA software. The dashed lines link *GRHL2* with the other six genes that are directly or indirectly *Grhl2*-mediated in mouse models. Gene roles pertaining to breast cancer function and disease are labeled in brown and purple respectively, and known clinical utility in green, using the Ingenuity® IPA software. Their therapeutic potential is given in **Table S3**. *GRHL2, CITED2* and *CTNNA3* are new markers according to Ingenuity database.
(TIF)

Figure S2 **Cross-species conserved regions among the predicted** ***Grh*** **-binding sites for genes** ***FN1*** **(A),** ***CITED2*** **(B),** ***CTNNB1*** **(C) and** ***CTNNA3*** **(D).**
(TIF)

Table S1 **Single and multivariable DMFS analysis for** ***GRHL2*** **and known prognostic factors.**
(DOC)

Table S2 **Prognostic marker candidates derived from literature and biological experiments to compare G+ cell lines with control cells.**
(DOC)

Table S3 **Evidence of therapeutic potential for the seven genes that construct the identified gene-set pair.**
(DOC)

Text S1 **Abbreviations.**
(DOC)

Data S1 **Prognostic tests of 2717 GSP combinations using three computational training cohorts with 995 patients.**
(XLS)

Data S2 **Prognostic indexes, the relative expression indexes of the identified GSP, of 388 independent validation patients in the pooled three-dataset.**
(PDF)
