# Supplementary figures and images for: Bridging Cancer Biology with the Clinic: Relative Expression of a GRHL2-Mediated Gene-Set Pair Predicts Breast Cancer Metastasis
Source: PLoS One. 2013 Feb 18;8(2):e56195. doi: 10.1371/journal.pone.0056195 (PMC3575392; doi:10.1371/journal.pone.0056195)

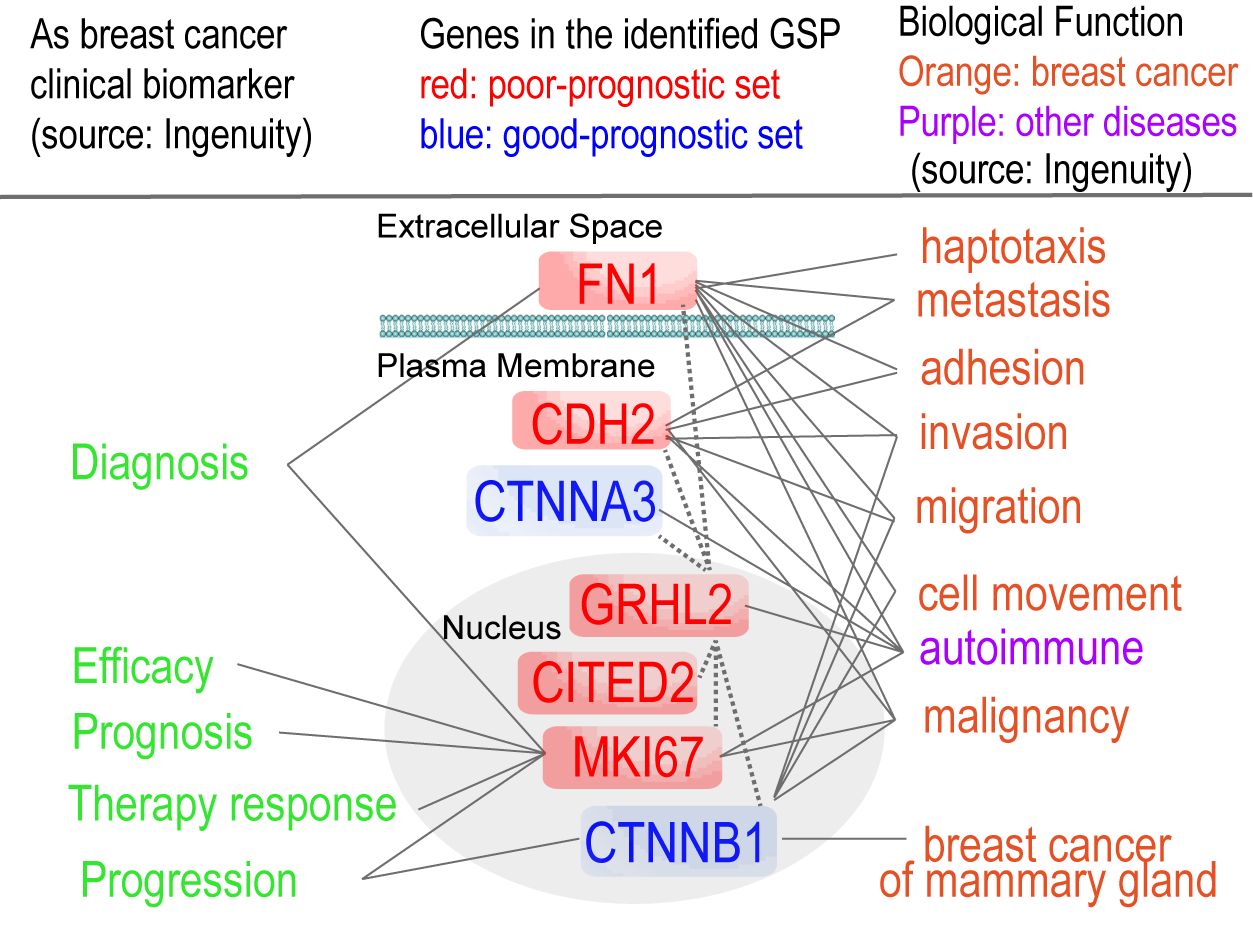

Supplement: Figure S1 — Clinical impacts of the seven genes in the identified GSP. The solid lines link genes with their clinical roles and biological functions, using the Ingenuity® IPA software. The dashed lines link GRHL2 with the other six genes that are directly or indirectly Grhl2-mediated in mouse models. Gene roles pertaining to breast cancer function and disease are labeled in brown and purple respectively, and known clinical utility in green, using the Ingenuity® IPA software. Their therapeutic potential is given in Table S3. GRHL2, CITED2 and CTNNA3 are new markers according to Ingenuity database. (TIF) [file pone.0056195.s001.tif]

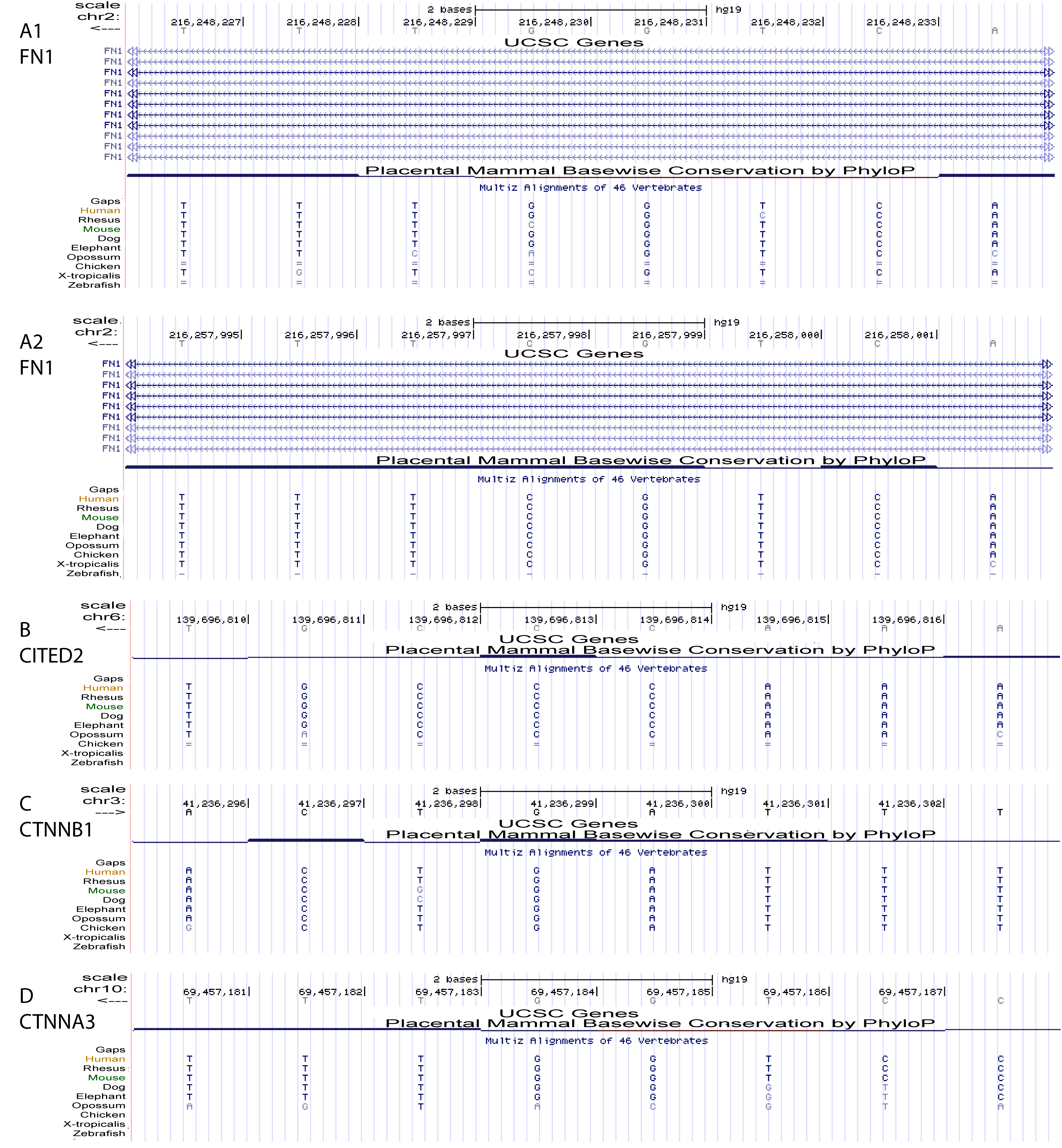

Supplement: Figure S2 — Cross-species conserved regions among the predicted Grh -binding sites for genes FN1 (A), CITED2 (B), CTNNB1 (C) and CTNNA3 (D). (TIF) [file pone.0056195.s002.tif]
