# Supplementary material for: Bridging Cancer Biology with the Clinic: Relative Expression of a GRHL2-Mediated Gene-Set Pair Predicts Breast Cancer Metastasis
Source: PLoS One. 2013 Feb 18;8(2):e56195. doi: 10.1371/journal.pone.0056195 (PMC3575392; doi:10.1371/journal.pone.0056195)
Supplement: Table S1 — Single and multivariable DMFS analysis for GRHL2 and known prognostic factors. (DOC) [file pone.0056195.s003.doc]

### Table S1. Single and multivariable distance metastasis-free survival analysis for *GRHL2* and known prognostic factors.

| **Variables** | 1. **Univariate analyses** | | | | 1. **Multivariate analyses** | | | |
| --- | --- | --- | --- | --- | --- | --- | --- | --- |
|  | **HR** | **95% CI** | **P Value** | **# of obs.** | **HR** | **95% CI** | **P Value** | **# of obs.** |
| *GRHL2* (log2 exp.) | 1.4 | 1.0-2.1 | **0.041** | 509 | 1.6 | 1.0-2.5 | **0.031** | 381 |
| ER (Pos. vs neg.) | 0.6 | 0.4-0.8 | **0.0009** | 504 | 0.6 | 0.4-0.9 | **0.027** | 381 |
| Lymph node (pos. vs neg.) | 1.7 | 1.2-2.4 | **0.0049** | 504 | 1.6 | 1.0-2.6 | **0.035** | 381 |
| Tumor size (>20mm) | 1.6 | 1.2-2.3 | **0.0059** | 506 | 1.5 | 1.0-2.3 | **0.049** | 381 |
| Grade (3 classes) | 1.4 | 1.0-1.9 | **0.03** | 391 | 1.1 | 0.8-1.5 | 0.75 | 381 |
| *GRHL2* (median) | 1.1 | 0.8-1.6 | 0.52 | 509 |  |  |  |  |
| Age (year) | 1.0 | 1.0-1.0 | 0.54 | 508 |  |  |  |  |

Cox proportional hazards analysis of (A) standard clinical factors alone, or (B) a multivariate model containing significant characters for the 947-sample combined set. ER status, lymph node status were a binary variable (1 = positive, 0 = negative); Tumor grade was a ternary variable (2 = grade III, 1 = grade II, 0=grade I); Tumor size was a binary variable (1=larger than 20mm, 0= less than or equal to 20mm). Variables found to be significant (p < 0.05) in the Cox proportional hazards model are shown in bold. HR: Hazard Ratio; CI: Confidence Interval; obs.: observation.

### 
