# Supplementary material for: Bridging Cancer Biology with the Clinic: Relative Expression of a GRHL2-Mediated Gene-Set Pair Predicts Breast Cancer Metastasis
Source: PLoS One. 2013 Feb 18;8(2):e56195. doi: 10.1371/journal.pone.0056195 (PMC3575392; doi:10.1371/journal.pone.0056195)
Supplement: Table S2 — Prognostic marker candidates derived from literature and biological experiments to compare G+ cell lines with control cells. (DOC) [file pone.0056195.s004.doc]

### Table S2. Prognostic marker candidates derived from literature and biological experiments to compare G+ cell lines with control cells. The starred genes construct the GSP with the capability to predict 1678 breast cancer metastases significantly which are further discussed in Table S3.

| **Symbol** | **Official gene name** | **Alteration in G+ cells** | **Impact on cancer metastasis** | **Ref.** |
| --- | --- | --- | --- | --- |
| ★*GRHL2* | grainyhead-like 2 (Drosophila) | up | In human breast cancers cell lines, *GRHL2* is amplified and overexpressed and is associated with a poor prognosis. In immunodeficient mice, *Grhl2* is significantly highly expressed in mice that initiated tumors and resistant to conventional chemotherapeutic reagents. |  |
| ★*CDH2* | cadherin 2, type 1, N-cadherin (neuronal) | up | Upregulated *CDH2* has been observed in invasive tumor cell lines and tissues from breast, prostate and melanomas. |  |
| *ACTA2* | actin, alpha 2, smooth muscle, aorta | up | Metastatic tumors showed significantly higher alpha-SMA expression, and tumors with high alpha-SMA expression have significantly poorer clinical outcomes. |  |
| ★*FN1* | fibronectin 1 | up | FN1 expression levels were significantly associated with DMFS in 680 lymph node-negative untreated patients (P<0.03). |  |
| *TGFB3* | transforming growth factor, beta 3 | up | *TGFb* expression prevents the apoptotic death of highly metastatic tumor cells in human and mouse breast cancer cell lines |  |
| ★*CITED2* | Cbp/p300-interacting transactivator, with Glu/Asp-rich carboxy-terminal domain, 2 | up* | *CITED2* expression is not only significantly associated with tumor aggressiveness, but also with clinical benefit from tamoxifen treatment. |  |
| ★*MKI67* | ki67 (Proliferation marker) | up^#^ | High *Ki67* expression is associated with larger tumor size, negative hormone receptor status, positive Her2 status, typical of aggressive tumors, and is an independently worse prognostic factor for breast cancer-specific death. |  |
| *RHOA* | ras homolog gene family, member A | up | In human breast cancer cell line, *RhoA* activation affects proliferation, migration, and tube formation, while *RhoA* knockdown inhibits these changes. |  |
| *CDH1* | cadherin 1, type 1, E-cadherin (epithelial) | down | E-cadherin negative deregulation is associated with metastatic potential in tumor cells due to increased invasively. |  |
| *CTNNA1* | catenin (cadherin-associated protein), alpha 1, 102kDa | down | Alpha- and beta-catenins are downregulated in invasive tumor cells and indicate a complete dissociation of the cadherin-catenin complex. |  |
| ★CTNNB1 | catenin (cadherin-associated protein), beta 1, 88kDa | down |
| ★*CTNNA3* | catenin (cadherin-associated protein), alpha 3 | down | *CTNNA3* together with the above two catenins increased the accuracy and sensitivity predicting metastasis and prognosis of breast cancer. |  |

#: Up-expressed in G+ MCF10 cell line, but down-expressed in G+ 4T1 and G+ MCF7 cell lines.

*: Instead of checking both mRNA and protein expression, only the mRNA expression was checked.

### References for Table S2

1. Dompe N, Rivers CS, Li L, Cordes S, Schwickart M, et al. (2011) A whole-genome RNAi screen identifies an 8q22 gene cluster that inhibits death receptor-mediated apoptosis. Proc Natl Acad Sci U S A 108: E943-951.

2. Leth-Larsen R, Terp MG, Christensen AG, Elias D, Kuhlwein T, et al. (2012) Functional heterogeneity within the CD44 high human breast cancer stem cell-like compartment reveals a gene signature predictive of distant metastasis. Mol Med.

3. Hazan RB, Qiao R, Keren R, Badano I, Suyama K (2004) Cadherin switch in tumor progression. Ann N Y Acad Sci 1014: 155-163.

4. Yamashita M, Ogawa T, Zhang X, Hanamura N, Kashikura Y, et al. (2012) Role of stromal myofibroblasts in invasive breast cancer: stromal expression of alpha-smooth muscle actin correlates with worse clinical outcome. Breast Cancer 19: 170-176.

5. Helleman J, Jansen MP, Ruigrok-Ritstier K, van Staveren IL, Look MP, et al. (2008) Association of an extracellular matrix gene cluster with breast cancer prognosis and endocrine therapy response. Clin Cancer Res 14: 5555-5564.

6. Hoshino Y, Katsuno Y, Ehata S, Miyazono K (2011) Autocrine TGF-beta protects breast cancer cells from apoptosis through reduction of BH3-only protein, Bim. J Biochem 149: 55-65.

7. van Agthoven T, Sieuwerts AM, Veldscholte J, Meijer-van Gelder ME, Smid M, et al. (2009) CITED2 and NCOR2 in anti-oestrogen resistance and progression of breast cancer. British journal of cancer 101: 1824-1832.

8. Kurebayashi J, Kanomata N, Shimo T, Yamashita T, Aogi K, et al. (2012) Marked lymphovascular invasion, progesterone receptor negativity, and high Ki67 labeling index predict poor outcome in breast cancer patients treated with endocrine therapy alone. Breast Cancer.

9. Yamamoto S, Ibusuki M, Yamamoto Y, Fu P, Fujiwara S, et al. (2012) Clinical relevance of Ki67 gene expression analysis using formalin-fixed paraffin-embedded breast cancer specimens. Breast Cancer.

10. Ma J, Zhao QL, Ren H, Liu WC, Xue Y (2012) [Role of RhoA in hypoxia-induced breast cancer cell VEGF secretion and proliferation, migration and tube formation of HUVECs]. Nan Fang Yi Ke Da Xue Xue Bao 32: 784-788.

11. Paredes J, Figueiredo J, Albergaria A, Oliveira P, Carvalho J, et al. (2012) Epithelial E- and P-cadherins: Role and clinical significance in cancer. Biochim Biophys Acta 1826: 297-311.

12. Morrogh M, Andrade VP, Giri D, Sakr RA, Paik W, et al. (2012) Cadherin-catenin complex dissociation in lobular neoplasia of the breast. Breast Cancer Res Treat 132: 641-652.

13. Zou W, Hu CH, Zhou JP (2002) [Relationship between the expression of E-cadherin-catenins and alpha-, beta-, gamma-catenin and the metastasis and prognosis of breast cancer]. Hunan Yi Ke Da Xue Xue Bao 27: 499-502.
