# Supplementary material for: Bridging Cancer Biology with the Clinic: Relative Expression of a GRHL2-Mediated Gene-Set Pair Predicts Breast Cancer Metastasis
Source: PLoS One. 2013 Feb 18;8(2):e56195. doi: 10.1371/journal.pone.0056195 (PMC3575392; doi:10.1371/journal.pone.0056195)
Supplement: Table S3 — Evidence of therapeutic potential for the seven genes that construct the identified gene-set pair. (DOC) [file pone.0056195.s005.doc]

### Table S3. Evidence of therapeutic potential for the seven genes that construct the identified gene-set pair.

| **Gene** | | **Breast cancer therapeutic potential** | **Reference** | |  |
| --- | --- | --- | --- | --- | --- |
| *GRHL2* | *Grhl2* suppresses the death-receptor and could serve as targets for therapeutics to increase tumor apoptosis. | | |  | |
| *CDH2* (N-cadherin) | *CDH2* is in an AP-2gamma directly regulated network that has therapeutic potential. Research shows N-cadherin can be inhibited by both progesterone and dexamethasone in endometrial cancer cells thus can be assessed in the future as a molecular target for new breast cancer therapies. | | |  | |
| *FN1* | *FN1* was reported as one of several potentially useful targets for breast cancer prognosis and therapy in 2003. Recently, *FN1* is found to be inhibited by miR-200c which extremely low in poorly differentiated cancer cells; and the restoration of miR-200c increases sensitivity to microtubule-targeting agents. | | |  | |
| *CITED2* | *CITED2* mRNA levels were predictive for a clinical benefit from first-line tamoxifen treatment in patients with advanced breast cancer; its induction also regulates colon cancer invasion, and can serve as a therapeutic target (through *HDAC* inhibitors). | | |  | |
| *MKI67* (ki67) | Recent studies have shown that *MKI67* is among six temporally therapy-response genes that characterize the response to endocrine therapy in breast tumors. Universally, *MKI67* expression can be reduced through the combination of everolimus and trastuzumab. | | |  | |
| *CTNNB1*(beta-catenin) | Adriamycin can induce E-cadherin-mediated cell-cell adhesion by increasing expression of E-cadherin and beta-catenin and decreasing expression of *MUC1* during breast cancer cell apoptosis induced by the drug. Beta-catenin also responds to the breast cancer suppression agent I3C. | | |  | |
| *CTNNA3* (γ-catenin) | Indole-3-carbinol (*I3C*), a compound found to suppress invasion and migration in breast cancer cells, significantly caused a dose-dependent increase in E-cadherin, three major catenins (alpha, beta, and gamma-catenin). | | |  | |

### References for Table S3

1. Dompe N, Rivers CS, Li L, Cordes S, Schwickart M, et al. (2011) A whole-genome RNAi screen identifies an 8q22 gene cluster that inhibits death receptor-mediated apoptosis. Proc Natl Acad Sci U S A 108: E943-951.

2. Ailan H, Xiangwen X, Daolong R, Lu G, Xiaofeng D, et al. (2009) Identification of target genes of transcription factor activator protein 2 gamma in breast cancer cells. BMC Cancer 9: 279.

3. Davies S, Dai D, Pickett G, Leslie KK (2006) Gene regulation profiles by progesterone and dexamethasone in human endometrial cancer Ishikawa H cells. Gynecol Oncol 101: 62-70.

4. Cochrane DR, Spoelstra NS, Howe EN, Nordeen SK, Richer JK (2009) MicroRNA-200c mitigates invasiveness and restores sensitivity to microtubule-targeting chemotherapeutic agents. Mol Cancer Ther 8: 1055-1066.

5. Mackay A, Jones C, Dexter T, Silva RL, Bulmer K, et al. (2003) cDNA microarray analysis of genes associated with ERBB2 (HER2/neu) overexpression in human mammary luminal epithelial cells. Oncogene 22: 2680-2688.

6. Bai L, Merchant JL (2007) A role for CITED2, a CBP/p300 interacting protein, in colon cancer cell invasion. FEBS Lett 581: 5904-5910.

7. van Agthoven T, Sieuwerts AM, Veldscholte J, Meijer-van Gelder ME, Smid M, et al. (2009) CITED2 and NCOR2 in anti-oestrogen resistance and progression of breast cancer. British journal of cancer 101: 1824-1832.

8. Taylor KJ, Sims AH, Liang L, Faratian D, Muir M, et al. (2010) Dynamic changes in gene expression in vivo predict prognosis of tamoxifen-treated patients with breast cancer. Breast Cancer Res 12: R39.

9. Kurebayashi J, Kanomata N, Shimo T, Yamashita T, Aogi K, et al. (2012) Marked lymphovascular invasion, progesterone receptor negativity, and high Ki67 labeling index predict poor outcome in breast cancer patients treated with endocrine therapy alone. Breast Cancer.

10. Zhu Y, Zhang X, Liu Y, Zhang S, Liu J, et al. (2012) Antitumor effect of the mTOR inhibitor everolimus in combination with trastuzumab on human breast cancer stem cells in vitro and in vivo. Tumour Biol.

11. Yang SZ, Kohno N, Kondo K, Yokoyama A, Hamada H, et al. (1999) Adriamycin activates E-cadherin-mediated cell-cell adhesion in human breast cancer cells. Int J Oncol 15: 1109-1115.

12. Meng Q, Qi M, Chen DZ, Yuan R, Goldberg ID, et al. (2000) Suppression of breast cancer invasion and migration by indole-3-carbinol: associated with up-regulation of BRCA1 and E-cadherin/catenin complexes. J Mol Med (Berl) 78: 155-165.
