# Supplementary material for: Bridging Cancer Biology with the Clinic: Relative Expression of a GRHL2-Mediated Gene-Set Pair Predicts Breast Cancer Metastasis
Source: PLoS One. 2013 Feb 18;8(2):e56195. doi: 10.1371/journal.pone.0056195 (PMC3575392; doi:10.1371/journal.pone.0056195)
Supplement: Text S1 — Abbreviations. (DOC) [file pone.0056195.s006.doc]

## List of abbreviations

RXA: relative expression analysis

GSP: gene-set pair

*GRHL2*: transcription factor grainy head-like 2

G+: *GRHL2* overexpressed

Sp: set of poor prognostic genes

Sg: set of good prognostic genes

M+: gene markers that had higher expression in G+ than in control cells

M-: gene markers that had lower expression in G+ than in control cells

EMT: epithelial-mesenchymal transitions

DMFS: distant metastasis-free survival

ATCC: American Type Culture Collection

shRNA: short hairpin RNA

ER: estrogen receptor

MDCK: Madin-Darby Canine Kidney Epithelial

PGR: progesterone receptors

FDR: false discovery rate
