# Supplementary material for: Bridging Cancer Biology with the Clinic: Relative Expression of a GRHL2-Mediated Gene-Set Pair Predicts Breast Cancer Metastasis
Source: PLoS One. 2013 Feb 18;8(2):e56195. doi: 10.1371/journal.pone.0056195 (PMC3575392; doi:10.1371/journal.pone.0056195)
Supplement: Data S2 — Prognostic indexes, the relative expression indexes of the identified GSP, of 388 independent validation patients in the pooled three-dataset. (PDF) [file pone.0056195.s008.pdf]

Bridging Cancer Biology to with the Clinic: Relative Expression of a GRHL2-mediated Gene-set Pair Predicts Breast Cancer Metastasis

Xinan “Holly” Yang, Prabhakaran Vasudevan, Vishwas Parekh, Aleks Penev, John M. Cunningham
Departments of Pediatrics, Section of Hematology/Oncology, and Comer Children’s Hospital, The University of Chicago

Data S2: Prognostic indexes (Is), the relative expression of the identified GSP, of 388 independent validation patients in the pooled three-dataset.

GEO sample ID	GSE Access ID	DMFS (year)	DMFS.event	Is>0
GSM308256	GSE12276	0.25	1	TRUE
GSM308257	GSE12276	2.08	1	TRUE
GSM308258	GSE12276	1.92	1	TRUE
GSM308259	GSE12276	1.08	1	TRUE
GSM308260	GSE12276	1.67	1	TRUE
GSM308261	GSE12276	1.50	1	TRUE
GSM308262	GSE12276	2.50	1	TRUE
GSM308263	GSE12276	5.75	1	TRUE
GSM308264	GSE12276	3.08	0	TRUE
GSM308265	GSE12276	1.92	1	TRUE
GSM308266	GSE12276	0.25	1	TRUE
GSM308267	GSE12276	1.42	1	TRUE
GSM308268	GSE12276	3.17	1	TRUE
GSM308269	GSE12276	0.67	1	TRUE
GSM308270	GSE12276	0.58	1	TRUE
GSM308271	GSE12276	2.42	0	TRUE
GSM308272	GSE12276	1.17	1	TRUE
GSM308273	GSE12276	1.58	1	TRUE
GSM308274	GSE12276	2.42	0	TRUE
GSM308275	GSE12276	0.58	1	TRUE
GSM308276	GSE12276	1.33	1	TRUE
GSM308277	GSE12276	1.83	1	TRUE
GSM308278	GSE12276	1.67	1	TRUE
GSM308279	GSE12276	1.67	1	TRUE
GSM308280	GSE12276	1.33	1	TRUE
GSM308281	GSE12276	0.42	1	TRUE
GSM308282	GSE12276	0.83	1	TRUE
GSM308283	GSE12276	1.08	1	TRUE
GSM308284	GSE12276	3.83	1	TRUE
GSM308285	GSE12276	0.58	1	TRUE
GSM308286	GSE12276	0.92	1	TRUE
GSM308287	GSE12276	3.50	1	TRUE
GSM308288	GSE12276	0.83	1	TRUE
GSM308289	GSE12276	5.08	1	TRUE
GSM308290	GSE12276	0.50	1	TRUE
GSM308291	GSE12276	2.08	1	TRUE
GSM308292	GSE12276	8.25	1	TRUE

GSM308293	GSE12276	0.42	1	TRUE
GSM308294	GSE12276	1.75	1	TRUE
GSM308295	GSE12276	0.92	1	TRUE
GSM308296	GSE12276	2.67	0	TRUE
GSM308297	GSE12276	1.17	1	TRUE
GSM308298	GSE12276	1.17	1	TRUE
GSM308299	GSE12276	1.50	1	TRUE
GSM308300	GSE12276	2.42	1	TRUE
GSM308301	GSE12276	1.25	0	TRUE
GSM308302	GSE12276	2.00	1	TRUE
GSM308303	GSE12276	3.25	1	TRUE
GSM308304	GSE12276	4.50	1	TRUE
GSM308305	GSE12276	1.25	1	TRUE
GSM308306	GSE12276	2.33	1	TRUE
GSM308307	GSE12276	2.42	1	TRUE
GSM308308	GSE12276	1.92	1	TRUE
GSM308309	GSE12276	1.67	1	TRUE
GSM308310	GSE12276	3.00	0	TRUE
GSM308311	GSE12276	3.00	1	TRUE
GSM308312	GSE12276	9.58	0	TRUE
GSM308313	GSE12276	0.33	1	TRUE
GSM308314	GSE12276	0.67	1	TRUE
GSM308315	GSE12276	5.00	1	TRUE
GSM308316	GSE12276	0.00	1	TRUE
GSM308318	GSE12276	3.50	1	TRUE
GSM308319	GSE12276	1.25	1	TRUE
GSM308320	GSE12276	0.58	1	TRUE
GSM308321	GSE12276	0.00	1	TRUE
GSM308322	GSE12276	2.25	1	TRUE
GSM308323	GSE12276	1.50	1	TRUE
GSM308324	GSE12276	1.17	1	TRUE
GSM308325	GSE12276	1.08	1	TRUE
GSM308326	GSE12276	1.58	1	TRUE
GSM308327	GSE12276	6.08	0	TRUE
GSM308328	GSE12276	1.33	1	TRUE
GSM308329	GSE12276	0.08	0	TRUE
GSM308330	GSE12276	0.00	1	TRUE
GSM308331	GSE12276	0.00	1	TRUE
GSM308332	GSE12276	3.17	1	TRUE
GSM308333	GSE12276	1.50	1	TRUE
GSM308334	GSE12276	1.92	0	TRUE
GSM308335	GSE12276	4.17	1	TRUE
GSM308336	GSE12276	0.67	1	TRUE
GSM308337	GSE12276	3.25	0	TRUE
GSM308338	GSE12276	0.83	1	TRUE
GSM308339	GSE12276	0.25	1	TRUE
GSM308340	GSE12276	5.00	1	TRUE

GSM308341	GSE12276	1.42	0	TRUE
GSM308342	GSE12276	1.92	1	TRUE
GSM308343	GSE12276	1.00	1	TRUE
GSM308344	GSE12276	0.50	1	TRUE
GSM308345	GSE12276	2.50	1	TRUE
GSM308346	GSE12276	1.08	1	TRUE
GSM308347	GSE12276	0.50	1	TRUE
GSM308348	GSE12276	2.08	1	TRUE
GSM308349	GSE12276	0.33	1	TRUE
GSM308350	GSE12276	1.25	1	TRUE
GSM308351	GSE12276	2.50	0	TRUE
GSM308352	GSE12276	0.00	1	TRUE
GSM308353	GSE12276	5.33	0	TRUE
GSM308354	GSE12276	1.75	1	TRUE
GSM308355	GSE12276	0.00	1	TRUE
GSM308356	GSE12276	1.50	1	TRUE
GSM308357	GSE12276	1.33	0	TRUE
GSM308358	GSE12276	0.00	1	TRUE
GSM308359	GSE12276	3.00	1	TRUE
GSM308360	GSE12276	3.25	1	TRUE
GSM308361	GSE12276	2.25	1	TRUE
GSM308362	GSE12276	1.25	1	TRUE
GSM308363	GSE12276	1.83	1	TRUE
GSM308364	GSE12276	2.42	1	TRUE
GSM308365	GSE12276	0.92	1	TRUE
GSM308366	GSE12276	4.83	1	TRUE
GSM308367	GSE12276	3.33	1	TRUE
GSM308368	GSE12276	4.00	1	TRUE
GSM308369	GSE12276	3.17	1	TRUE
GSM308370	GSE12276	1.00	1	TRUE
GSM308371	GSE12276	1.08	1	TRUE
GSM308372	GSE12276	7.08	1	TRUE
GSM308373	GSE12276	0.75	1	TRUE
GSM308374	GSE12276	0.50	1	TRUE
GSM308375	GSE12276	2.33	1	TRUE
GSM308376	GSE12276	0.50	1	TRUE
GSM308377	GSE12276	1.58	1	TRUE
GSM308378	GSE12276	1.25	1	TRUE
GSM308380	GSE12276	0.58	0	TRUE
GSM308381	GSE12276	9.58	1	TRUE
GSM308382	GSE12276	3.83	1	TRUE
GSM308383	GSE12276	0.75	1	TRUE
GSM308384	GSE12276	2.67	1	TRUE
GSM308385	GSE12276	2.25	1	TRUE
GSM308386	GSE12276	0.42	1	TRUE
GSM308387	GSE12276	0.33	1	TRUE
GSM308388	GSE12276	1.42	0	TRUE

GSM308389	GSE12276	1.25	1	TRUE
GSM308391	GSE12276	0.58	1	TRUE
GSM308393	GSE12276	2.75	1	TRUE
GSM308394	GSE12276	1.83	1	TRUE
GSM308398	GSE12276	1.00	1	TRUE
GSM308399	GSE12276	3.17	1	TRUE
GSM308401	GSE12276	1.08	1	TRUE
GSM308404	GSE12276	0.67	1	TRUE
GSM308406	GSE12276	4.83	0	TRUE
GSM308407	GSE12276	7.83	1	TRUE
GSM308408	GSE12276	0.33	1	TRUE
GSM308409	GSE12276	0.00	0	TRUE
GSM308410	GSE12276	3.17	1	TRUE
GSM308413	GSE12276	4.08	1	TRUE
GSM308414	GSE12276	8.08	1	TRUE
GSM308415	GSE12276	4.17	1	TRUE
GSM308416	GSE12276	4.42	1	TRUE
GSM308417	GSE12276	1.92	1	TRUE
GSM308418	GSE12276	2.08	1	TRUE
GSM308419	GSE12276	1.83	1	TRUE
GSM308420	GSE12276	3.00	1	TRUE
GSM308421	GSE12276	2.92	1	TRUE
GSM308422	GSE12276	1.33	1	TRUE
GSM308423	GSE12276	2.92	1	TRUE
GSM308424	GSE12276	1.92	1	TRUE
GSM308425	GSE12276	1.17	1	TRUE
GSM308426	GSE12276	0.75	1	TRUE
GSM308427	GSE12276	3.50	1	TRUE
GSM308428	GSE12276	1.42	1	TRUE
GSM308429	GSE12276	0.42	1	TRUE
GSM308430	GSE12276	4.58	1	TRUE
GSM308431	GSE12276	1.92	1	TRUE
GSM308432	GSE12276	3.33	1	TRUE
GSM308433	GSE12276	3.67	1	TRUE
GSM308434	GSE12276	3.92	1	TRUE
GSM308435	GSE12276	2.17	1	TRUE
GSM308436	GSE12276	1.75	1	TRUE
GSM308437	GSE12276	2.92	1	TRUE
GSM308438	GSE12276	2.92	1	TRUE
GSM308439	GSE12276	0.33	1	TRUE
GSM308440	GSE12276	0.42	1	TRUE
GSM308441	GSE12276	3.50	1	TRUE
GSM308442	GSE12276	1.50	1	TRUE
GSM308443	GSE12276	1.17	1	TRUE
GSM308444	GSE12276	1.33	1	TRUE
GSM308445	GSE12276	2.50	1	TRUE
GSM308446	GSE12276	3.50	1	TRUE

GSM308447	GSE12276	2.00	1	TRUE
GSM308448	GSE12276	2.08	1	TRUE
GSM308449	GSE12276	3.92	1	TRUE
GSM308450	GSE12276	2.75	1	TRUE
GSM308451	GSE12276	2.42	1	TRUE
GSM308452	GSE12276	2.08	1	TRUE
GSM308453	GSE12276	6.92	1	TRUE
GSM308454	GSE12276	6.08	1	TRUE
GSM308455	GSE12276	2.58	1	TRUE
GSM308456	GSE12276	2.33	1	TRUE
GSM308457	GSE12276	1.50	1	TRUE
GSM308458	GSE12276	0.75	1	TRUE
GSM308459	GSE12276	0.58	1	TRUE
GSM308460	GSE12276	8.92	1	TRUE
GSM491175	GSE19615	2.00	1	TRUE
GSM491176	GSE19615	3.17	0	TRUE
GSM491177	GSE19615	5.00	0	TRUE
GSM491178	GSE19615	0.75	1	TRUE
GSM491179	GSE19615	5.17	0	TRUE
GSM491180	GSE19615	1.33	1	TRUE
GSM491181	GSE19615	5.33	0	FALSE
GSM491182	GSE19615	5.58	0	TRUE
GSM491183	GSE19615	2.25	1	TRUE
GSM491184	GSE19615	5.17	0	TRUE
GSM491185	GSE19615	4.33	0	TRUE
GSM491186	GSE19615	0.08	1	TRUE
GSM491187	GSE19615	4.42	0	TRUE
GSM491188	GSE19615	4.50	0	TRUE
GSM491189	GSE19615	4.25	0	TRUE
GSM491190	GSE19615	4.00	0	TRUE
GSM491191	GSE19615	4.33	0	TRUE
GSM491192	GSE19615	4.17	0	TRUE
GSM491193	GSE19615	4.17	0	TRUE
GSM491194	GSE19615	3.33	0	TRUE
GSM491195	GSE19615	4.08	0	TRUE
GSM491196	GSE19615	4.42	0	TRUE
GSM491197	GSE19615	4.17	0	TRUE
GSM491198	GSE19615	2.92	1	TRUE
GSM491199	GSE19615	4.17	0	TRUE
GSM491200	GSE19615	3.00	0	TRUE
GSM491201	GSE19615	3.00	0	TRUE
GSM491202	GSE19615	3.25	0	TRUE
GSM491203	GSE19615	5.42	0	TRUE
GSM491204	GSE19615	6.67	0	FALSE
GSM491205	GSE19615	6.50	0	TRUE
GSM491206	GSE19615	6.50	0	TRUE
GSM491207	GSE19615	4.25	0	TRUE

GSM491208	GSE19615	0.50	1	TRUE
GSM491209	GSE19615	7.08	0	TRUE
GSM491210	GSE19615	6.08	0	TRUE
GSM491211	GSE19615	6.83	0	TRUE
GSM491212	GSE19615	6.25	0	TRUE
GSM491213	GSE19615	6.58	0	FALSE
GSM491214	GSE19615	4.33	0	TRUE
GSM491215	GSE19615	6.75	0	TRUE
GSM491216	GSE19615	4.92	0	TRUE
GSM491217	GSE19615	6.92	0	TRUE
GSM491218	GSE19615	7.08	0	TRUE
GSM491219	GSE19615	1.50	1	TRUE
GSM491220	GSE19615	5.83	0	FALSE
GSM491221	GSE19615	6.92	0	TRUE
GSM491222	GSE19615	6.17	0	TRUE
GSM491223	GSE19615	7.25	0	TRUE
GSM491224	GSE19615	7.17	0	TRUE
GSM491225	GSE19615	7.17	0	TRUE
GSM491226	GSE19615	6.67	0	FALSE
GSM491227	GSE19615	6.50	0	TRUE
GSM491228	GSE19615	6.08	0	TRUE
GSM491229	GSE19615	4.67	0	TRUE
GSM491230	GSE19615	5.83	0	TRUE
GSM491231	GSE19615	6.92	0	TRUE
GSM491232	GSE19615	1.17	1	TRUE
GSM491233	GSE19615	6.25	0	TRUE
GSM491234	GSE19615	6.42	0	FALSE
GSM491235	GSE19615	6.08	0	TRUE
GSM491236	GSE19615	4.92	0	TRUE
GSM491237	GSE19615	7.08	0	TRUE
GSM491238	GSE19615	6.42	0	TRUE
GSM491239	GSE19615	6.75	0	TRUE
GSM491240	GSE19615	6.75	0	FALSE
GSM491241	GSE19615	4.75	0	TRUE
GSM491242	GSE19615	4.00	0	FALSE
GSM491243	GSE19615	4.83	0	FALSE
GSM491244	GSE19615	1.75	1	FALSE
GSM491245	GSE19615	6.42	0	TRUE
GSM491246	GSE19615	6.83	0	TRUE
GSM491247	GSE19615	5.92	0	TRUE
GSM491248	GSE19615	4.58	0	TRUE
GSM491249	GSE19615	0.17	1	TRUE
GSM491250	GSE19615	6.33	0	TRUE
GSM491251	GSE19615	7.33	0	TRUE
GSM491252	GSE19615	3.92	0	TRUE
GSM491253	GSE19615	6.92	0	FALSE
GSM491254	GSE19615	4.50	0	TRUE

GSM491255	GSE19615	6.58	0	TRUE
GSM491256	GSE19615	1.25	1	TRUE
GSM491257	GSE19615	1.50	1	TRUE
GSM491258	GSE19615	6.33	0	FALSE
GSM491259	GSE19615	6.75	0	FALSE
GSM491260	GSE19615	6.92	0	FALSE
GSM491261	GSE19615	4.50	0	TRUE
GSM491262	GSE19615	6.58	0	TRUE
GSM491263	GSE19615	6.17	0	TRUE
GSM491264	GSE19615	6.17	0	TRUE
GSM491265	GSE19615	6.75	0	FALSE
GSM491266	GSE19615	6.75	0	TRUE
GSM491267	GSE19615	6.33	0	TRUE
GSM491268	GSE19615	5.42	0	TRUE
GSM491269	GSE19615	6.92	0	TRUE
GSM491270	GSE19615	4.00	0	TRUE
GSM491271	GSE19615	4.25	0	TRUE
GSM491272	GSE19615	4.67	0	TRUE
GSM491273	GSE19615	4.92	0	TRUE
GSM491274	GSE19615	5.75	0	TRUE
GSM491275	GSE19615	5.92	0	TRUE
GSM491276	GSE19615	4.42	0	TRUE
GSM491277	GSE19615	4.42	0	TRUE
GSM491278	GSE19615	4.42	0	TRUE
GSM491279	GSE19615	4.50	0	TRUE
GSM491280	GSE19615	5.92	0	FALSE
GSM491281	GSE19615	4.00	0	FALSE
GSM491282	GSE19615	5.92	0	FALSE
GSM491283	GSE19615	5.92	0	TRUE
GSM491284	GSE19615	6.17	0	TRUE
GSM491285	GSE19615	3.00	0	FALSE
GSM491286	GSE19615	1.50	1	FALSE
GSM491287	GSE19615	6.08	0	TRUE
GSM491288	GSE19615	5.58	0	TRUE
GSM491289	GSE19615	4.58	0	TRUE
GSM50072	GSE2063	10.74	0	TRUE
GSM50073	GSE2063	7.25	0	TRUE
GSM50094	GSE2063	3.22	1	TRUE
GSM50074	GSE2063	7.32	0	FALSE
GSM50075	GSE2063	7.32	0	TRUE
GSM50095	GSE2063	6.54	1	FALSE
GSM50096	GSE2063	1.38	1	FALSE
GSM50107	GSE2063	6.21	0	FALSE
GSM50034	GSE2063	7.25	0	TRUE
GSM50097	GSE2063	5.39	1	FALSE
GSM50078	GSE2063	6.98	0	TRUE
GSM50098	GSE2063	2.57	1	TRUE

GSM50035	GSE2063	1.84	1	TRUE
GSM50079	GSE2063	6.52	0	FALSE
GSM50108	GSE2063	3.96	0	FALSE
GSM50110	GSE2063	5.19	0	TRUE
GSM50111	GSE2063	7.30	0	FALSE
GSM50112	GSE2063	1.18	1	FALSE
GSM50114	GSE2063	1.30	1	FALSE
GSM50115	GSE2063	4.42	0	FALSE
GSM50116	GSE2063	3.58	0	FALSE
GSM50118	GSE2063	6.28	0	TRUE
GSM50119	GSE2063	4.18	0	TRUE
GSM50120	GSE2063	3.68	0	TRUE
GSM50121	GSE2063	4.12	0	TRUE
GSM50122	GSE2063	3.24	1	TRUE
GSM50123	GSE2063	3.90	0	TRUE
GSM50127	GSE2063	5.24	0	TRUE
GSM50059	GSE2063	7.63	0	FALSE
GSM50060	GSE2063	3.47	1	TRUE
GSM50061	GSE2063	3.96	1	FALSE
GSM50062	GSE2063	3.07	1	TRUE
GSM50063	GSE2063	3.11	1	FALSE
GSM50064	GSE2063	0.94	1	FALSE
GSM50065	GSE2063	5.81	0	TRUE
GSM50066	GSE2063	1.24	1	FALSE
GSM50128	GSE2063	2.30	0	TRUE
GSM50067	GSE2063	0.77	1	TRUE
GSM50068	GSE2063	3.48	1	TRUE
GSM50036	GSE2063	9.15	0	TRUE
GSM50037	GSE2063	6.86	0	FALSE
GSM50038	GSE2063	6.47	0	FALSE
GSM50039	GSE2063	7.08	0	TRUE
GSM50040	GSE2063	7.57	0	FALSE
GSM50042	GSE2063	4.41	0	FALSE
GSM50043	GSE2063	5.23	0	TRUE
GSM50044	GSE2063	5.62	0	TRUE
GSM50045	GSE2063	10.75	0	TRUE
GSM50046	GSE2063	8.04	0	TRUE
GSM50047	GSE2063	5.41	0	FALSE
GSM50048	GSE2063	6.07	0	TRUE
GSM50130	GSE2063	3.36	0	TRUE
GSM50049	GSE2063	5.50	0	TRUE
GSM50069	GSE2063	3.83	1	TRUE
GSM50051	GSE2063	3.77	0	FALSE
GSM50052	GSE2063	4.56	0	FALSE
GSM50053	GSE2063	3.97	0	TRUE
GSM50054	GSE2063	4.05	0	FALSE
GSM50080	GSE2063	7.25	0	TRUE

GSM50081	GSE2063	5.07	0	TRUE
GSM50106	GSE2063	1.58	1	TRUE
GSM50082	GSE2063	7.11	0	FALSE
GSM50099	GSE2063	1.30	1	TRUE
GSM50083	GSE2063	7.28	0	TRUE
GSM50084	GSE2063	6.40	0	TRUE
GSM50085	GSE2063	6.50	0	FALSE
GSM50086	GSE2063	8.31	0	FALSE
GSM50087	GSE2063	8.31	0	FALSE
GSM50100	GSE2063	5.81	1	FALSE
GSM50089	GSE2063	8.74	0	FALSE
GSM50131	GSE2063	4.59	1	TRUE
GSM50090	GSE2063	6.79	0	FALSE
GSM50091	GSE2063	7.19	0	TRUE
GSM50101	GSE2063	6.36	1	TRUE
GSM50092	GSE2063	6.45	0	TRUE
GSM50102	GSE2063	0.67	1	FALSE
GSM50103	GSE2063	1.84	1	TRUE
GSM50104	GSE2063	6.77	1	FALSE
GSM50093	GSE2063	7.86	0	FALSE
GSM50070	GSE2063	3.36	1	TRUE
GSM50105	GSE2063	7.19	0	TRUE
